# Strength, faith, and vision

**DOI:** 10.3325/cmj.2011.52.743

**Published:** 2011-12

**Authors:** Nika Matovac

Dear readers,

I am Nika Matovac, a medical student and daughter of Vesna Andrijević-Matovac, who was diagnosed with breast cancer when I was only 11 years old. As you might know, when my mum was writing this column, she was talking about her feelings, her fears, her tears, she told you stories from her life and the life of her friends who were in the same situation… I’ve written about how I felt during all these years, how it looks like when a woman with breast cancer goes on holidays, and now I am asking myself what to write about, and who I am to talk about such a delicate situation as is that of women with breast cancer. Therefore, I’ve decided to tell you a few stories that revolve around a single basic idea – HELP THOSE IN NEED.

My mum was born on Christmas day, December 25, 1960 as the first child of her parents, Nada and Berislav. She was a very lively child, but was often afraid for her health. My mum met my dad at the age of 18 and at that time she was a really good violinist, future student of economics, and truly big hypochondriac. I was born when my mum was 29 as the first and only child. Before my mum’s illness, life was really a fairytale. She would make the best birthday parties and the most beautiful gingerbread house you could imagine, she would buy me everything I wanted, she had the most beautiful smile, was a better driver than some men, sang beautifully, and made her world turn around mine – she changed her job just to be more at home with me, cooked me lunch, drove me to school, went shopping with me (and did not show the bill to my dad, cause that would be a disaster). We were mum and daughter, but what’s even better we were best friends. If you ask yourself what changed after she had got her diagnosis on October 8, 2001, the answer is – honestly, not much. After that day, my mum was fighting even harder to be with me, to see me growing, to see me becoming a woman, but it was not easy.

I am a medical student and I’ve wanted to become a doctor since I was a child. I’ve always been fascinated with needles, hospitals, medicine, and the idea of how to help and cure people, especially babies, because they are just beginning their life. I’ve just started my 4th year, and two days ago, one of our teachers asked us if we had ever been taught how to deliver bad news to patients. The answer was unfortunately NO, no one had taught us that. I have to say that we were lucky to meet such a teacher, because nothing is harder than to tell someone that they have to struggle hard, go through a difficult medical treatment, and going to die…

My wish is to become a pediatrician, but I already know because I have learned it from my family, especially my mum, that helping others helps ourselves, because we feel better, we feel useful, and that’s why I must promise that in the future I will volunteer even more in “EVERYTHING for HER!” and other associations.

The association for women diagnosed with and treated for breast cancer, their families, and friends “EVERYTHING for HER!”, was established on March 21, 2008 by three patients: my mum, Vesna Andrijević-Matovac, Almenka Balenović, and Bojana Benović. This is a humanitarian, voluntary, and non-profit organization with the goal of providing informative, psychological, and logistical assistance to women suffering from breast and other cancers. The association established the Center for Psychological Assistance in Zagreb. The Center offers several types of psychotherapy, such as individual, partnership, family psychological support, group psychological treatments, and support groups, as well as some educational workshops on nutrition, physiotherapy, special types of yoga and relaxation techniques, and other anti-stress techniques. Also the Center provides accommodation for women coming to chemotherapy from other parts of Croatia or other countries. All services provided by the Center are free of charge. The Center is there for those who need it and you wouldn’t believe how many people need it.

I could repeat what I’ve written here for days, because I want to show you what a woman with the strength, faith, and vision can make. These three patients who established “EVERYTING for HER!” gave their soul and opened their heart to the others who needed such support and help. I dare to call them heroes, because despite their illness they found the strength to listen to others, to support others, to make others feel better and to make them not feel alone. Now, my dear readers, when you know what only three women have done, how they have changed the life of so many women and how they will continue to change the lives of many others through this Center for psychological support, just imagine what we could do together, so, especially in this Christmas time, let’s HELP THOSE IN NEED.

